# The Expanding Point of Care Ultrasound (POCUS) Paradigm

**DOI:** 10.24908/pocusj.v10i01.18408

**Published:** 2025-04-15

**Authors:** Katie Wiskar

**Affiliations:** 1 Department of Medicine, University of British Columbia, Vancouver, CAN

**Keywords:** ultrasound, education, clinical decision-making, ultrasound policy

Point of Care Ultrasound (POCUS) is an ever-evolving technology that has become integral to clinical practice in a variety of domains. Since its inception in Emergency Medicine (EM) in the 1980s, POCUS has traditionally been viewed as a tool to make binary decisions in response to focused clinical questions [1,2]. Current literature continues to cite POCUS as a means to rule in or out specific diagnoses and answer simple, yes-or-no questions: *Is there a live intrauterine pregnancy? Is this abdominal pain caused by an abdominal aortic aneurysm? Is this leg swelling due to a deep vein thrombosis* [3–5]? The adage that each POCUS scan should answer one specific question is often recited in POCUS education tools, and POCUS archiving and reporting materials are typically centered around this same binary paradigm [6]. This view is particularly prevalent among those who are less familiar with POCUS, who view the tool as a limited, operator-dependent technology to make these types of quick decisions.

To be sure, these types of single-system, dichotomous scans have their place, and continue to be an important part of POCUS in certain settings. However, as the field expands and evolves, so too must our conceptualization of POCUS and the paradigm in which it is used. POCUS is increasingly applied in multi-organ assessments that answer complex clinical questions: *How can I improve the hemodynamics of this patient in shock? What is the best strategy for fluid management in this multimorbid patient? What factors are contributing to respiratory failure in this hypoxic patient?* These types of assessments are multifaceted. They often incorporate scans from multiple organ systems, utilize advanced Doppler parameters when available, and integrate POCUS data points with other hemodynamic and clinical parameters [7–9]. They may be repeated serially in response to changes in clinical status or therapeutic interventions [10]. They may not provide a simple, dichotomous answer to a clinical question. Instead, they provide data points that help shape diagnostic and therapeutic thinking, with layers of nuance and complexity that depends on the skill and experience of the operator. POCUS functions in the same way as physical exam findings or lab tests, which may impact our clinical decisions via Bayesian reasoning rather than by definitely offering a single diagnosis [11].

The mental model in which we frame POCUS is paramount as the technology becomes increasingly embedded in undergraduate and postgraduate medical training programs [12,13]. As POCUS leaders, we have a responsibility to ensure that the younger generation of medical learners understands both the expanded potential of POCUS as well as the nuances and caveats that accompany a non-dichotomous approach. Though only a select few practitioners will develop advanced POCUS expertise, an understanding of how the technology can properly be deployed is beneficial even to novice-level users.

Assuredly, this expanded POCUS model remains distinct from radiology-performed imaging or traditional consultative echocardiography. Rather than an exhaustive examination of all the involved organs, POCUS fundamentally derives from a clinical question. The extent of the POCUS examination will therefore depend on the clinical situation, as well as expertise of the operator. Studies looking at cursory whole-body screening POCUS exams in unselected patients have shown limited benefit [14]. Furthermore, integration with other clinical data points remains paramount – POCUS itself provides the clinical correlation ubiquitously cited in radiology reports.

While an expansion beyond the traditional binary model of POCUS introduces more nuance and room for clinical judgement, it also introduces more potential for error. The classic dichotomous EM-based model has at its center a focus on safety and ruling out life-threatening diagnoses. Scans with excellent negative predictive value therefore became dominant [1]. As we move beyond this exclusive domain, we are able to incorporate techniques with less robust test metrics. These still provide useful information if interpreted in the correct clinical context. Nuanced, multi-layered POCUS assessments demand a solid understanding of the underlying anatomic, physiologic, and hemodynamic variables at play; otherwise, this opens up room for misinterpretation and error [15]. Core tenets of being aware of one's own limitations are essential to safely utilizing this powerful technology [16].

The paradigm for POCUS use in the modern era must evolve. As the lowest-hanging fruit, single-system exams directed at binary clinical questions will continue to have their place. However, even these should always be performed with a careful understanding of their limitations and thoughtful integration with other data points. We must make room for an expanded vision of what POCUS can entail, including answering complex, nuanced questions with multi-organ assessment. POCUS is an indispensable clinical tool in the right hands and we must be prepared to use that tool to its full potential.

## References

[1] Osterwalder J, Polyzogopoulou E, Hoffmann B. Point-of-Care Ultrasound—History, Current and Evolving Clinical Concepts in Emergency Medicine. Med. 2023;59(12):1–16. 10.3390/medicina59122179 PMC10744481 38138282

[2] Jehle D, Guarino J, Karamanoukian H. Emergency ultrasound in the evaluation of blunt abdominal trauma. Am J Emerg Med. 1993;11(4):342–6. 8216513 10.1016/0735-6757(93)90164-7

[3] Choi W, Cho YS, Ha YR, Oh JH, Lee H, Kang BS, et al. Role of point-of-care ultrasound in critical care and emergency medicine: update and future perspective. Clin Exp Emerg Med. 2023;10(4):363–81. 38225778 10.15441/ceem.23.101 PMC10790072

[4] Hashim A, Tahir MJ, Ullah I, Asghar MS, Siddiqi H, Yousaf Z. The utility of point of care ultrasonography (POCUS). Ann Med Surg. 2021;71(November):102982. 10.1016/j.amsu.2021.102982 PMC8606703 34840746

[5] Lewis D, Rang L, Kim D, Robichaud L, Kwan C, Pham C, et al. Recommendations for the use of point-of-care ultrasound (POCUS) by emergency physicians in Canada. Can J Emerg Med. 2019;21(6):721–6. 10.1017/cem.2019.392 31771691

[6] Chelikam N, Vyas A, Desai R, Khan N, Raol K, Kavarthapu A, et al. Past and Present of Point-of-Care Ultrasound (PoCUS): A Narrative Review. Cureus. 2023;15(12). 10.7759/cureus.50155 PMC10771967 38192958

[7] Millington SJ, Wiskar K, Hobbs H, Koenig S. Risks and Benefits of Fluid Administration as Assessed by Ultrasound. Chest. 2021;160(6):2196–208. 34245742 10.1016/j.chest.2021.06.041

[8] Narasimhan M, Koenig SJ, Mayo PH. A Whole-Body Approach to Point of Care Ultrasound. Chest. 2016;150(4):772–6. 27568582 10.1016/j.chest.2016.07.040

[9] Morosanu B, Balan C, Boros C, Dazzi F, Wong A, Corradi F, et al. Incidence, predictability, and outcomes of systemic venous congestion following a fluid challenge in initially fluid-tolerant preload-responders after cardiac surgery: a pilot trial. Crit Care. 2024;28(1):339. 39439007 10.1186/s13054-024-05124-6 PMC11494747

[10] Torres-Arrese M, de Casasola-Sánchez GG, Méndez-Bailón M, Montero-Hernández E, Cobo-Marcos M, Rivas-Lasarte M, et al. Usefulness of Serial Multiorgan Point-of-Care Ultrasound in Acute Heart Failure: Results from a Prospective Observational Cohort. Med. 2022;58(1). 10.3390/medicina58010124 PMC8780545 35056432

[11] Ashby D, Smith AFM. Evidence-based medicine as Bayesian decision-making. Stat Med. 2000;19(23):3291–305. 11113960 10.1002/1097-0258(20001215)19:23<3291::aid-sim627>3.0.co;2-t

[12] Hoppmann RA, Mladenovic J, Melniker L, Badea R, Blaivas M, Montorfano M, et al. International consensus conference recommendations on ultrasound education for undergraduate medical students. Ultrasound J. 2022;14(1):1–32. 35895165 10.1186/s13089-022-00279-1 PMC9329507

[13] Ramgobin D, Gupta V, Mittal R, Su L, Patel MA, Shaheen N, et al. POCUS in Internal Medicine Curriculum: Quest for the Holy-Grail of Modern Medicine. J Community Hosp Intern Med Perspect. 2022;12(5):36–42. 10.55729/2000-9666.1112 PMC9529654 36262489

[14] Weile J, Frederiksen CA, Laursen CB, Graumann O, Sloth E, Kirkegaard H. Point-of-care ultrasound induced changes in management of unselected patients in the emergency department - A prospective single-blinded observational trial. Scand J Trauma Resusc Emerg Med. 2020;28(1):1–9. 32471452 10.1186/s13049-020-00740-x PMC7260768

[15] Kazory A, Olaoye OA, Koratala A. Nuances of Point-of-Care Ultrasound in Nephrology: A Clarion Call for Deeper Understanding. Blood Purif. 2024;53(7):598–602. 38621364 10.1159/000538909

[16] Blanco P, Volpicelli G. Common pitfalls in point-of-care ultrasound: a practical guide for emergency and critical care physicians. Crit Ultrasound J. 2016;8(1). 10.1186/s13089-016-0052-x PMC5081982 27783380

